# Nitrogen-driven stem elongation in poplar is linked with wood modification and gene clusters for stress, photosynthesis and cell wall formation

**DOI:** 10.1186/s12870-014-0391-3

**Published:** 2014-12-30

**Authors:** Dejuan Euring, Hua Bai, Dennis Janz, Andrea Polle

**Affiliations:** Forest Botany and Tree Physiology, Georg-August Universität Göttingen, Büsgenweg 2, 37077 Göttingen, Germany

**Keywords:** Development, Metaxylem, Nitrogen use, *Populus trichocarpa*, Stress, Transcriptome, Wood, Xylem

## Abstract

**Background:**

Nitrogen is an important nutrient, often limiting plant productivity and yield. In poplars, woody crops used as feedstock for renewable resources and bioenergy, nitrogen fertilization accelerates growth of the young, expanding stem internodes. The underlying molecular mechanisms of nitrogen use for extension growth in poplars are not well understood. The aim of this study was to dissect the nitrogen-responsive transcriptional network in the elongation zone of *Populus trichocarpa* in relation to extension growth and cell wall properties.

**Results:**

Transcriptome analyses in the first two internodes of *P. trichocarpa* stems grown without or with nitrogen fertilization (5 mM NH_4_NO_3_) revealed 1037 more than 2-fold differentially expressed genes (DEGs). Co-expression analysis extracted a network containing about one-third of the DEGs with three main complexes of strongly clustered genes. These complexes represented three main processes that were responsive to N-driven growth: Complex 1 integrated growth processes and stress suggesting that genes with established functions in abiotic and biotic stress are also recruited to coordinate growth. Complex 2 was enriched in genes with decreased transcript abundance and functionally annotated as photosynthetic hub. Complex 3 was a hub for secondary cell wall formation connecting well-known transcription factors that control secondary cell walls with genes for the formation of cellulose, hemicelluloses, and lignin. Anatomical and biochemical analysis supported that N-driven growth resulted in early secondary cell wall formation in the elongation zone with thicker cell walls and increased lignin. These alterations contrasted the N influence on the secondary xylem, where thinner cell walls with lower lignin contents than in unfertilized trees were formed.

**Conclusion:**

This study uncovered that nitrogen-responsive elongation growth of poplar internodes is linked with abiotic stress, suppression of photosynthetic genes and stimulation of genes for cell wall formation. Anatomical and biochemical analysis supported increased accumulation of cell walls and secondary metabolites in the elongation zone. The finding of a nitrogen-responsive cell wall hub may have wider implications for the improvement of tree nitrogen use efficiency and opens new perspectives on the enhancement of wood composition as a feedstock for biofuels.

**Electronic supplementary material:**

The online version of this article (doi:10.1186/s12870-014-0391-3) contains supplementary material, which is available to authorized users.

## Background

Woody biomass is a valuable resource for the generation of renewable energy and an important feedstock for fiber, pulp and cellulose production [1-3]. It is formed during the process of secondary growth. The molecular regulation of secondary growth is intensively being studied in poplar and in the model plant *Arabidopsis thaliana* [4-9]. For example, cell differentiation in the vascular cambium is determined by auxin, auxin transporters, and auxin-responsive transcription factors [7,10]. Furthermore, transcriptional regulation involves members of the *AUXIN RESPONSE FACTOR (ARF), MYB, NAC*, and *WRKY* gene families [11-14] whose interplay eventually determines the amounts of cellulose, hemicellulose, and lignin produced during secondary cell wall formation [7].

The prerequisite for secondary growth is primary growth and shoot elongation. The molecular regulation of cell division and differentiation have mainly been addressed in *Arabidopsis* [15,16]. In the shoot apical meristem the transcription factors *WUSCHEL* (*WUS*) [17], *CLAVATA* (*CLV*), *SHOOT MERISTEMLESS* (*STM*) [18], and *KNOX* [19] have been identified as key actors in the control of the size of stem cell population and production of new cell files. They are regulated by hormones, like cytokinins, gibberellin and auxin [20]. Gradients of auxin and signaling peptides are important during the early steps of vascular development [7]. During primary growth, proto- and metaxylem elements are formed. Their differentiation is controlled by transcription factors of the VND (*VASCULAR-RELATED NAC DOMAIN*) family, *VND7* and *VND6* [21]. VNDs regulate down-stream transcription factors, especially MYB46 which plays a major role for the orchestration of biosynthetic genes for secondary cell wall formation [22-26]. Although primary growth that drives the elongation of the newly formed internodes is as important for wood production as secondary growth, very little is known about the molecular regulation underlying these developmental processes in poplars.

With regard to yield improvement, molecular links between primary growth and nitrogen (N) are of particular interest. Low N frequently limits productivity and consequently, fertilization can enhance yield [27]. Increased N availability results in enhanced leaf area production, increased photosynthesis and higher stem biomass production in poplars [28,29]. However, the wood of fertilized poplars is often characterized by thinner cell walls, less lignification, and increased amounts of tension wood [30-35]. In the developing xylem, key transcription factors for wood formation such as *WKRY* and *NAC* domain factors were decreased in hybrid poplars exposed to high (7.5 mM NH_4_NO_3_) compared with those grown with adequate N supply (0.75 mM NH_4_NO_3,_ [36]). Furthermore, the expression levels of several genes involved in hemicellulose and lignin biosynthesis were also reduced, while cellulose synthase increased under high compared with adequate N [36]. The observed transcriptional changes matched alterations in cell wall properties, for example the shift to lower lignin and higher cellulose concentrations in the wood of fertilized compared with non-fertilized poplars [36]. In contrast to radial growth, the influence of N on gene regulation during stem elongation has not been investigated. It is unknown whether high N mainly accelerates primary growth processes such as extension or whether it also impacts on cell wall properties. Understanding the molecular mechanisms of plant N usage for increased wood production and the consequences for wood properties is urgently needed.

In this study, we analyzed the genome-wide transcriptional responses to N fertilization in the elongation zone (EZ) of *P. trichocarpa.* We conducted co-expression analysis to establish networks of signaling, regulatory and functional genes underlying N-responsive stem growth. We dissected three main regulatory complexes that represent phytohormone-related development, regulation of photosynthesis and cell wall formation as the main processes underlying N-driven elongation growth. Because the transcriptional analysis predicted stimulation of the secondary metabolism in the EZ of N fertilized compared to unfertilized poplars, lignin and phenolic concentrations were also determined.

## Methods

### Plant material, growth conditions and treatment

**Figure 1:**
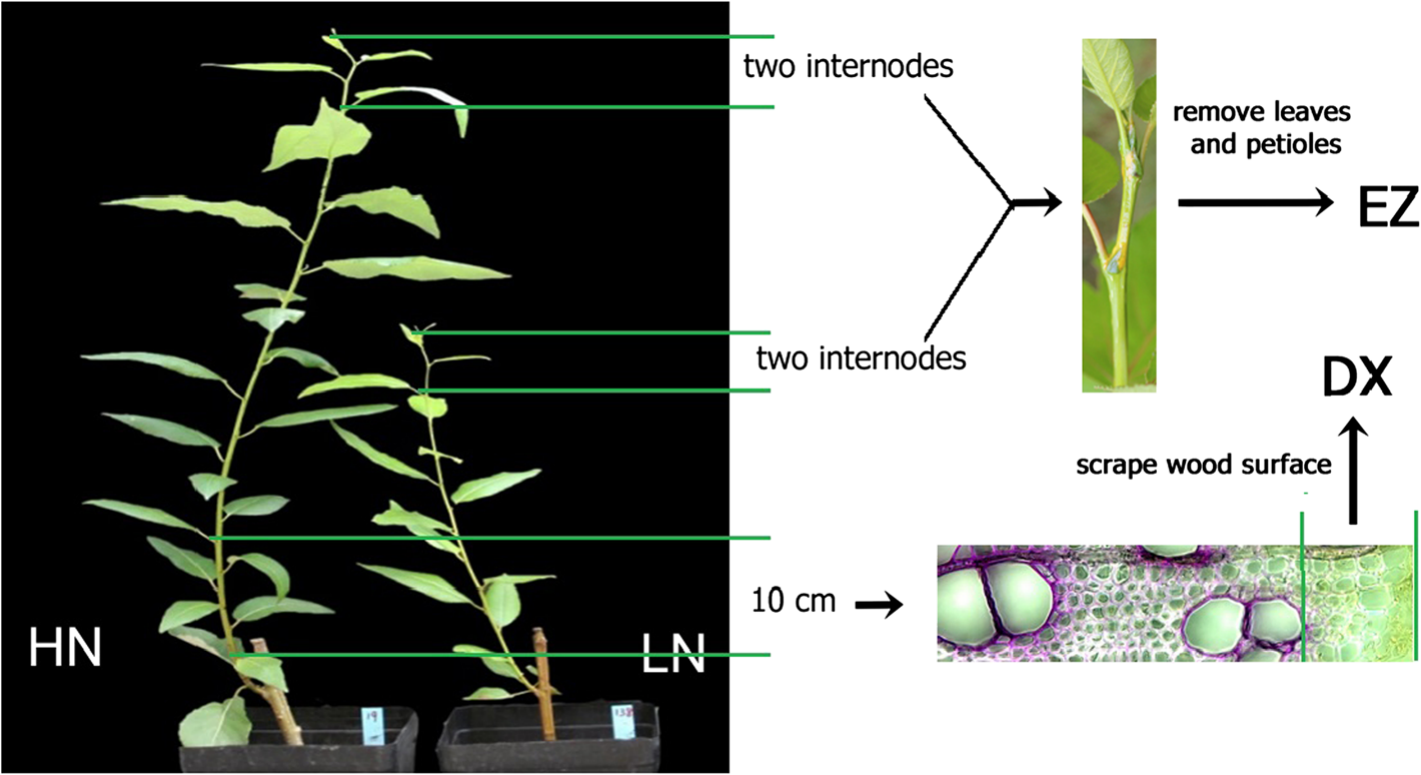
**Performance of poplars (** ***P. trichocarpa*** **) after 6 week of growth without or with addition of 5 mM NH**_**4**_ **NO**_**3**_ **.** Stem positions used for sample collection are indicated.

Twenty-four *Populus trichocarpa*, cultivar Weser 6 (Kompetenzzentrum HessenRohstoffe, Germany) cuttings were planted in 5 L pots with 20% compost soil and 80% sand at the end of April, 2009. The cuttings were cultivated in a greenhouse for 2 months under long-day conditions (16 hours light from 6:00 a.m. to 10:00 p.m) with a photosynthetically active radiation (PAR) of 150 μmol · m^−2^ · s^−1^ (fluorescent lamps L58W/25 and 58 W/840, Osram, Munich, Germany, and TLD 58 W/840 Philips, Amsterdam, Netherlands). Afterwards, the plants were divided into two groups, of similar average height. One group was fertilized with 120 ml 5 mM NH_4_NO_3_ (HN); the other group received the same amount of tap water (LN). Both groups were irrigated twice a week for 1.5 months. At the harvest, the height and basal stem diameter were measured with a folding ruler and a caliper (Tchibo GmbH, Hamburg, Germany). The total fresh weight of leaves, stems and roots were weighed. Two to three centimeter-long basal stem segments were stored in FAE (2% (v/v) formaldehyde, 5% (v/v) acetic acid, 63% (v/v) ethanol). Preliminary analyses of growth showed that elongation was confined to the first 5 to 7 internodes. Here, the first two internodes from the top including shoot apex were harvested and called elongation zone (EZ). Developing xylem was harvested from a 10 cm long stem segment at the bottom (Figure 1). The surface of debarked wood was scraped with a razor blade as described previously [37,38]. The samples, which consisted of a soft mush of tissue, were shock-frozen in liquid nitrogen and stored at −80°C. Aliquots of fresh leaves, stems and roots were weighed, oven dried at 60°C for 2 weeks and weighed again. Total dry biomass of leaves, stems and roots were calculated as: dry mass of aliquot × whole plant tissue fresh mass/fresh mass of the aliquot.

### Anatomical analyses

The second internodes counted from the top and bottom stem segments were fixed in FAE for one week, and transferred into plastic bottles with 70% ethanol for several days. Cross-sections (50 μm) were obtained with a sliding microtome (Reichert-Jung, Heidelberg, Germany). The sections were stained with Wiesner reagent (5.25 g phloroglucinol, 350 ml 95% ethanol, 175 ml 25% HCl) for 3 min [39] and mounted in 50% glycerol for microscopy. Sections were immediately viewed under a light microscope (Axioplan, Zeiss, Oberkochen, Germany) and photographed with 400-fold magnification using a digital camera (AxioCamMR3, Zeiss, Oberkochen, Germany). The image analysis software Image J (http://rsbweb.nih.gov/ij/; NIH, Bethesda, Maryland, USA) was used to measure the thickness of the double fiber walls and vessel lumen areas.

Cross sections of 10-μm thickness from the second internode counted from the top and bottom stem segments (liquid nitrogen shock frozen samples) were obtained with a cryo-microtome (Reichert-Jung, Model 2800 Frigocut N, Leica Instruments GmbH, Nussloch, Germany). The sections were immediately mounted in 50% glycerol for microscopy. Sections were immediately viewed under UV light (filters: BP 546, FT580, LP590) with a light microscope (Axioplan, Zeiss, Oberkochen, Germany) and photographed with 400-fold magnification using a digital camera (AxioCamMR3, Zeiss, Oberkochen, Germany). Phenolic compounds showed blue and chloroplasts red fluorescence. Defined areas of 1000 μm^2^ were selected to count chloroplasts.

### Lignin, phenolics and nitrogen analyses

To measure phenolics, frozen plant tissues were ground in a ball mill (Retsch, Haan, Germany). Fine powder (60 mg per sample) was extracted with 2 ml of 50% methanol in an ultrasonic bath (60 min, 40°C; Sonorex Super RK 510 H, Bandelin electronics, Berlin, Germany). The extract was centrifuged, the pellet was extracted once again in the dark in 2 ml of 50% methanol at room temperature for 60 min and the supernatants were combined for photometrical analysis of soluble phenolics with the Folin Ciocalteus method [31]. Catechin (Sigma-Aldrich, Deisenhofen, Germany) was measured to create a calibration curve and the phenolic concentrations were expressed as catechin equivalents.

Dry plant tissues were milled to a fine powder (MM2 Retsch, Haan, Germany) for the determination of lignin and nitrogen concentrations. To determine lignin, one to four mg dry powder materials were mixed with 25% acetyl bromide in acetic acid. The reaction tubes were incubated at 70°C for 30 min with shaking at 10 min intervals. After digestion, 250 μl sodium hydroxide (2 M) was added. After mixing, the reaction tubes were centrifuged with 15000 × g for 5 min at 4°C. The supernatant (138 μl) was added to new reaction tubes with 2.8 μl hydroxylamine (0.5 M) and 1.25 ml acetic acid (96%). A concentration series of coniferyl alcohol, analyzed with the same procedure as the analytical samples, was done to create a standard curve. The absorbance of the resulting solutions was measured at 280 nm after [40].

To determine nitrogen concentrations, aliquots of 0.7 - 0.9 mg dry milled powder were weighed (Sartorius Supermicro S4, Göttingen, Germany) into tin capsules (Hekatech, Wegberg, Germany) and analyzed in an Elemental Analyzer EA1108 (Carlo Erba Strumentazione, Rodano, Italy). Acetanilide (71.09% C, 10.36% N; Carlo Erba Strumentazione) was the standard.

Independent two-sample *t*-tests were carried out in Microsoft Excel to test whether means were significantly different at P < 0.05.

### RNA extraction and cDNA preparation

Shock frozen tissue of the EZ was ground in a pre-cooled ball mill (Retsch, Hann, Germany). Total RNA was extracted from 1 g tissue powder using hexadecyltrimethylammonium bromide extraction protocol [41]. The quantity and quality of total RNA were determined with a spectrophotometer (BioPhotometer, Eppendorf, Hamburg, Germany) by determining the ratio of absorbance of the sample at 260 nm to that of 280 nm. To remove DNA, 10 μg preparations was treated with DNase (Turbo DNA-free kit, Ambion, Austin, TX) at 37°C for 30 min according to the manufacturer’s instructions of Turbo DNA-free kit. DNase-treated total RNA (5 μg) was used as starting material for double-stranded cDNA synthesis using Oligo(dT)_18_ primer and RevertAid™ First Strand cDNA Synthesis Kit (MBI Fermentas, St. Leon-Rot, Germany) according to the manual.

### Microarrays and data analysis

Two biological samples of EZ were pooled. Three independent samples (representing 6 plants) of total RNA were prepared for whole-genome Affymetrix GeneChip microarray analysis. The quality of RNA was examined by MFTServices (Tübingen, Germany). WT-Ovation Pico RNA Amplification System (NuGen, San Carlos, CA) was used to amplify 50 ng total RNA to produce labeled cDNA. Six cDNA sets were hybridized to Poplar Genome Arrays (three arrays for LN and 3 arrays for HN plants) according to the manufacturer’s protocol (Affymetrix, Santa Clara, CA, USA). The microarray data set supporting the results of this article is available under the ArrayExpress accession number E-MTAB-1483, http://www.ebi.ac.uk/arrayexpress/experiments/E-MTAB-1483/. Gene expression analysis was performed with R Project software package, version 2.10.1 (http://www.R-project.org). cDNA Microarray data were normalized across the six arrays using Bioconductor - Robust Multiarray Averaging (RMA). Transcription levels of HN plants were compared to LN plants. Genes with var < 0.5 was removed. Significance Analysis of Microarrays (SAM) was performed to calculate *p*-values. Differentially expressed genes (DEGs) with fold change ≥ 2 and *p*-value ≤ 0.05 after Benjamini-Hochberg correction were annotated using Poparray (http://aspendb.uga.edu/poparray) for JGI poplar gene models and predicted *Arabidopsis* homologs. The differentially presented Gene Ontology (GO) categories were identified in Popgenie v3.0 (http://popgenie.org/) using the Analysis Tool GO enrichment. Enrichment analysis of MapMan categories [42] was conducted with Superviewer (http://bar.utoronto.ca/ntools/cgi-bin/ntools_classification_superviewer.cgi) calculating the mean and SD for 100 bootstraps of the input set (duplicates allowed) and the p of the hypergeometric distribution [43]. Gene coexpression relationships were calculated for the DEGs with the Analysis Tool PopNet in Popgenie v3.0 (http://popgenie.org/) with a display threshold of 7 and an expand threshold of 3. The coexpression analysis was based on microarray data from 21 experiments (GSE12152, GSE13109, GSE13990, GSE15242, GSE15595, GSE16420, GSE16459, GSE16495, GSE16786, GSE16888, GSE17223, GSE17225, GSE17226, GSE17230, GSE17804, GSE19279, GSE19467, GSE20061, GSE21061, GSE21171, GSE9673) which are studies using poplar and analyzing wood formation, growth, development, and the responses to nitrogen limitation and drought (http://popgenie.org/). Gene co-expression relationships were visualized in Cytoscape 3.1.1 [44]. Sub-clusters (= complexes) in networks were identified with Cytocluster applying the NonOverlapping algorithm and complex size threshold 3 (http://apps.cytoscape.org/apps/cytocluster).

## Results

### Nitrogen accelerates stem elongation and biomass production

*P. trichocarpa* plants were grown either without additional N (LN) or supplied with 5 mM NH_4_NO_3_ (HN). The fertilized poplars showed 1.4 times faster stem elongation rates than non-fertilized plants (Table 1) resulting in taller plants after 6 weeks of N treatment (Figure 1). N-induced growth stimulation also resulted in about 20% thicker stem diameter and almost doubled stem biomass compared to non-fertilized plants (Table 1). All stem tissues of HN poplars contained higher N concentrations than those of LN plants (Table 1).

**Table 1:** **Growth, biomass and nitrogen concentrations of** ***Populus trichocarpa***

**Parameter**	**LN**	**HN**	**P**
Height increment (cm d^−1^ )	0.83 ± 0.02	1.16 ± 0.03	<0.001
Stem diameter (mm)	5.35 ± 0.15	6.40 ± 0.14	<0.001
Biomass of stem (g plant^−1^)	2.31 ± 0.09	4.06 ± 0.17	<0.001
Biomass of leaves (g plant^−1^)	4.43 ± 0.24	7.17 ± 0.35	<0.001
Biomass of roots (g plant^−1^)	1.45 ± 0.13	1.67 ± 0.24	0.215
Total biomass (g plant^−1^)	8.49 ± 0.43	13.26 ± 0.64	<0.001
N in EZ (%)	2.03 ± 0.08	3.70 ± 0.15	<0.001
N in developing xylem (%)	0.55 ± 0.03	1.59 ± 0.08	<0.001
N in wood (%)	0.18 ± 0.02	0.42 ± 0.10	0.033
N in bark (%)	0.38 ± 0.06	1.03 ± 0.16	0.004

*Populus trichocarpa* were grown under low (LN) and high (HN) nitrogen conditions. Data are means ± SE of 12 biological replicates. Independent two-sample *t*-tests were used to test the differences between the means of HN and LN plants. Height increment was determined during the period of N fertilization. Stem diameter and dry biomass were determined at plant harvest.

### N-responsive stem elongation growth at the transcriptional level

To investigate the molecular basis of the N-accelerated elongation growth, transcriptome analyses were conducted in the EZ of HN and LN poplars. N fertilization resulted in 1037 differentially expressed genes (DEGs based on Populus v3Best Gene Models corresponding to 1208 Affymetrix IDs) with more than 2-fold significantly changed transcript levels in the EZ (Additional file 1: Table S1). GO term enrichment analysis revealed 24 significant Plant GO Slim categories for the N-responsive DEGs in the EZ (Table 2). From this list we deduced that processes related to cell wall formation were particularly prominent because we found GO terms related to “cell death” and “carbohydrate metabolism” in the category of biological processes and terms indicative for the extracellular compartment such as “cell wall”, “external encapsulating structure” and “proteinaceous extracellular matrix” in the category “cellular component”. In the category molecular function, the terms “kinase activity”, “signal transducer activity”, “RNA binding” and “receptor activity” pointed to signaling and transcriptional regulation as major activities in the EZ (Table 2).

**Table 2:** **Significantly enriched gene ontology (GO) terms in the elongation zone of** ***Populus trichocarpa***

**GO identity**	**Category**	**Description**	***P*** **value (FDR)**	**ClusterFreq**	**TotalFreq**
GO:0019538	P	protein metabolic process	<0.001	73/494	3727/14903
GO:0009987	P	cellular process	<0.001	227/494	8214/14903
GO:0006412	P	Translation	0.002	5/494	567/14903
GO:0006464	P	cellular protein modification process	0.003	47/494	2227/14903
GO:0008219	P	cell death	0.013	8/494	605/14903
GO:0016265	P	Death	0.013	8/494	605/14903
GO:0005975	P	carbohydrate metabolic process	0.018	50/494	998/14903
GO:0003824	F	catalytic activity	<0.001	401/618	10616/19350
GO:0005515	F	protein binding	<0.001	92/618	4123/19350
GO:0005198	F	structural molecule activity	<0.001	3/618	548/19350
GO:0005488	F	Binding	0.004	352/618	12290/19350
GO:0016301	F	kinase activity	0.011	46/618	2150/19350
GO:0003676	F	nucleic acid binding	0.017	72/618	3014/19350
GO:0000166	F	nucleotide binding	0.019	112/618	4388/19350
GO:0004871	F	signal transducer activity	0.023	5/618	448/19350
GO:0003723	F	RNA binding	0.035	6/618	461/19350
GO:0004872	F	receptor activity	0.060	3/618	298/19350
GO:0005840	C	Ribosome	0.004	2/162	469/6012
GO:0016020	C	Membrane	0.012	59/162	1584/6012
GO:0030312	C	external encapsulating structure	0.014	10/162	142/6012
GO:0005618	C	cell wall	0.014	10/162	142/6012
GO:0005576	C	extracellular region	0.025	5/162	52/6012
GO:0005622	C	Intracellular	0.026	17/162	1144/6012
GO:0005578	C	proteinaceous extracellular matrix	0.035	3/162	22/6012

Plant GOSlim terms were analyzed in Popgenie v3.0 (http://popgenie.org/) using the Analysis Tool GO enrichment (*P* value < 0.05, FDR adjusted). The input was the list of genes with significantly changed transcript levels in response to fertilization with 5 mM NH_4_NO_3_ for 1.5 month compared with non-fertilized plants. GO terms are indicated for biological processes (P), molecular functions (F) and cellular components (C). ClusterFreq and TotalFreq indicate the number of genes for a GO term found in the sample set and in the total genome, respectively.

### An N-responsive gene network in the elongation zone

**Figure 2:**
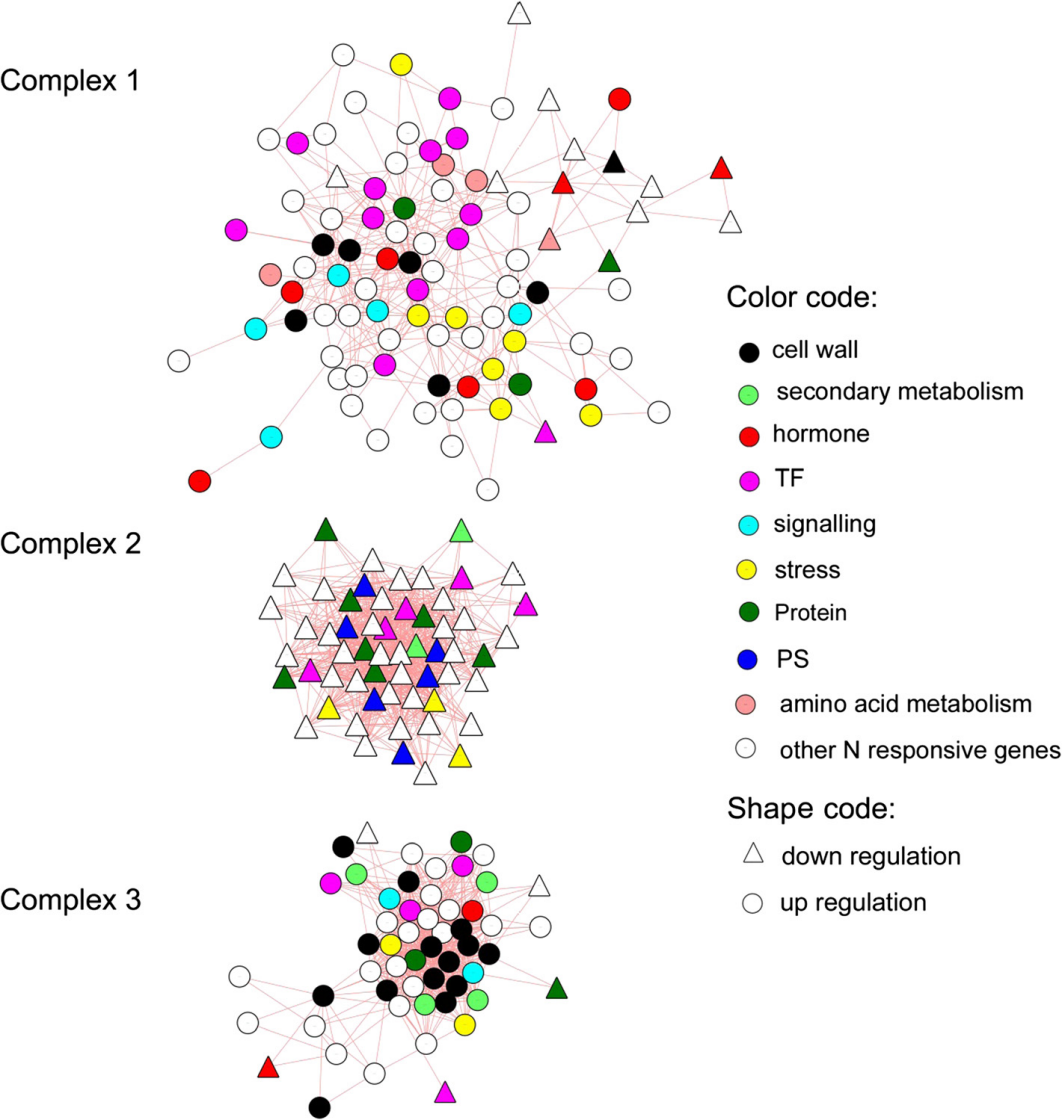
**Three main complexes of an N-responsive network in the elongation zone of** ***P*** **.** ***trichocarpa.*** Gene-gene relationships were obtained in Popgenie v3.0 (http://popgenie.org/) using the PopNet tool. The network was visualized in Cytoscape.

To find key pathways for the N regulation of elongation growth, the 1037 N-responsive DEGs in the EZ of the poplar stem were investigated with the PopNet tool in Popgenie v3.0. This analysis resulted in an N-responsive network with 392 nodes (= genes) connected by 1863 edges (= significant co-expression relationships) (Additional file 2: Table S2, Additional file 3: Figure S1). When the network was dissected into its subclusters, fourteen complexes (subclusters) were obtained. While most of these complexes were small consisting only of 3 to 12 nodes that were connected by 2 to 23 edges, three main complexes were retrieved: complex 1 (92 nodes, 364 edges), complex 2 (57 nodes, 740 edges) and complex 3 (54 nodes, 547 edges) (Figure 2). These complexes were also clearly apparent in the original network, in which complex 2 and complex 3 were connected through complex 1 (Additional file 3: Figure S1).

**Figure 3:**
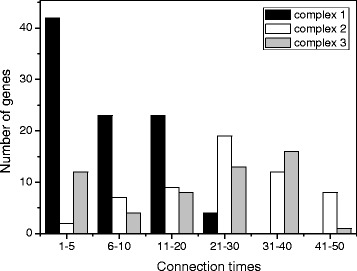
**Histogram of the number of neighbors for the genes in the main complexes of an N-responsive gene network in the elongation zone of** ***P*** **.** ***trichocarpa.*** The histogram classifies the number of edges in the three complexes and revealed differences for the distribution of genes in complex 1 compared with complex 2 or complex 3.

The clustering coefficients of complex 2 (0.73) and complex 3 (0.82) were higher than that of complex 3 (0.51) (Table 3). Average connectivity of a node in the complex 2 and complex 3 were more than 20 neighbours, while about 8 neighbours were present for a node in complex 1 (Table 3). A histogram of the edges per node revealed the maximum numbers of genes in the class of 21 to 30 for complex 2 and 31 to 40 edges for complex 3 underpinning the strong connectivity of these complexes (Figure 3). In complex 1 the highest node connectivity was 21 to 30 edges per node, but the maximum number of nodes was in the category with the lowest connectivity (1 to 5 edges per node) (Figure 3). The genes with the highest connectivity included a homolog to *PAD4*, a lipase involved in plant immune response and fitness [45] in complex 1 (26 edges), a scarcely defined nucleotide binding protein with functions in chloroplast metabolism [46] in complex 2 (48 edges) and a cellulose synthase [47] in complex 3 (37 edges) (Additional file 4: Table S3).

**Table 3:** **Network characteristics of three identified main complexes**

**Parameter**	**Complex 1**	**Complex 2**	**Complex 3**
Clustering coefficient	0.51	0.73	0.82
Net diameter	7	3	4
Net radius	4	2	2
Net centrality	0.20	0.41	0.41
Shortest path	8372	3192	2862
Characteristic path length	2.85	1.56	1.80
Mean number of neighbors	7.9	26.0	20.3
Nodes	92	57	54
Net density	0.09	0.46	0.38
Net heterogeneity	0.74	0.46	0.63
Edges	364	740	547
Number of Arabidopsis matches in the nodes	90	57	52

Networks were generated in Popgenie v3.0 (http://popgenie.org/) with the significantly regulated genes in the elongation zone of *P. trichocarpa*. Network parameters were calculated with Cytoscape.

**Figure 4:**
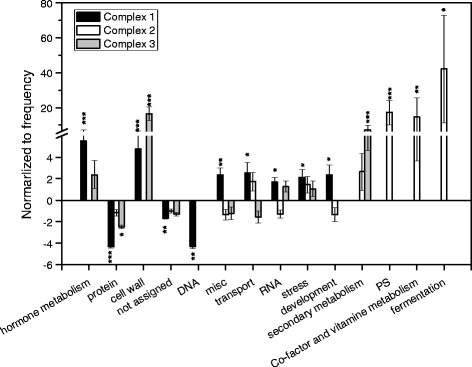
**Significantly enriched and depleted MapMan categories in the main complexes of an N-responsive gene network in the elongation zone of** ***P*** **.** ***trichocarpa.*** Genes identified in main complexes of the N-responsive network of the poplar elongation zone were assigned to the best *Arabidopsis* matches (AGI numbers) using Poparray (http://aspendb.uga.edu/poparray) and categorized with Superviewer. Data are means ± SD for 100 bootstraps of the initial data set. Stars indicate: *p < 0.05, **p < 0.01, ***p < 0.001.

To investigate whether the three complexes represent functional units we conducted Mapman analyses for the genes in each complex. A total of 14 significant categories for the DEGs in complex 1, complex 2 and complex 3 of the N-responsive network were identified (Figure 4). Complex 1 was enriched in the categories “hormone metabolism”, mainly because of several auxin-related genes, and further genes related to other plant growth hormones (gibberellin-, jasmonate- brassinosteroide- and ethylene-related genes), “development” (NAC factors *ANAC047* and *ANAC*061, Late embryogenesis abundant hydroxyproline-rich glycoprotein family), “RNA” with transcription factors related to stress and development (*WRKY18, WRKY26, WRKY33, WRKY40, HB07*), “stress”, mainly with drought stress-related genes such as *DREB, OSMOTIN, DnaJ, PAD4, ZAT10*)” and “transport” (nucleotide-sugar transporter, ABC type transporters and amino acid transporters) (Figure 4, Additional file 2: Table S2).

Both complex 1 and complex 3 were enriched in the category “cell wall”, but with genes indicating divergent functions in the two complexes. In complex 1, genes encoding proteins for hemicellulose and pectin metabolism were enriched (e.g. arabinogalactan protein 26, SNF1-related protein kinase, β-xylosidase 1, xyloglucan endotransglucosylase/hydrolase 15, xyloglucan endotransglycosylase 6, expansin). In complex 3, “cell wall” and “secondary metabolism” genes typically involved in the formation of cellulose (cellulose synthases *IRX1, IRX 3, IRX5*) and phenolic compounds including lignin (peroxidases, laccases, pinoresinol reductase) such as*, IRX 6, IRX 8, IRX 9, IRX 12, IRX 15,* and related transcription factors *MYB46* and *MYB56* were overrepresented (Figure 4, Additional file 2: Table S2).

Complex 2 showed a compositional pattern that differed strongly from complex 1 and 3 with significant enrichments in the categories “photosynthesis” (some nuclear encoded genes for light reaction and Calvin cycle)”, “co-factor and vitamin metabolism” (thiazole and thiamin production) and “fermentation” (aldehyde dehydrogenase) (Figure 4). It was furthermore notable that most of the DEGs in complex 2 were suppressed, whereas those in the complexes 1 and 3 showed in increased transcript abundance. Counting of chloroplasts in selected areas of the cross sections suggested that the suppression of photosynthetic genes was not linked with a reduction in the number of chloroplasts (35 ± 13 chloroplasts per 1000 μm^2^ and 27 ± 12 chloroplasts per 1000 μm^2^ , P = 0.220) in HN compared with LN poplars.

### N fertilization increases the concentrations of lignin and phenolic compounds in the poplar elongation zone

**Figure 5:**
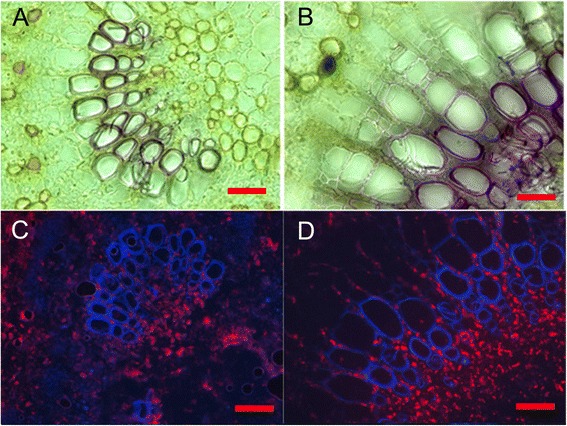
**Cross sections of the elongation zone of** ***P*** **.** ***trichocarpa*** **in response to low nitrogen (A, C) and high nitrogen (B, D) supply.** Lignin staining **(A, B)** of cross sections (50 μm thickness); autofluorescence of phenolics **(C, D)** in cross sections (10 μm thickness). The cross sections were taken in the second internode counted from the stem apex. The scale bars correspond to 50 μm.

Because genes related to secondary metabolism were overrepresented in complex 3, we tested whether the concentrations of lignin or soluble phenolic concentrations differed between the EZ from HN and LN poplars. The EZ of HN poplars contained higher concentrations of lignin and phenolics than that of LN poplars (Table 4). Cross sections that were stained for lignin supported an increased production of primary xylem with strong incorporation of lignin and secondary compounds in HN grown poplars compared with LN poplars (Figure 5).

**Table 4:** **Chemical and anatomical characteristics of stem tissues of** ***P. trichocarpa*** **grown without (LN) or with nitrogen fertilization** **(HN) for 1.5 months**

**Tissue**	**Parameter**	**LN**	**HN**	**p**
EZ	Lignin (%)	17.61 ± 1.50	21.28 ± 0.78	0.037
	Soluble phenolics (μg mg^−1^)	5.96 ± 0.52	7.89 ± 0.54	0.022
	Lumen per vessel (μm^2^)	189.0 ± 13.0	478.1 ± 32.9	<0.001
	Cell wall thickness of vessels (μm)	1.80 ± 0.15	2.20 ± 0.13	0.021
	FW/DW	5.10 ± 0.18	5.51 ± 0.06	0.035
Developing Xylem	Lignin (%)	11.43 ± 1.02	8.45 ± 1.33	0.063
	Soluble phenolics (μg mg^−1^)	0.86 ± 0.13	0.55 ± 0.08	0.045
	Lumen per vessel (μm^2^)	221.0 ± 32.5	600.6 ± 51.2	0.008
	Cell wall thickness of vessels (μm)	1.89 ± 0.12	1.74 ± 0.14	0.212
	FW/DW	3.15 ± 0.42	5.17 ± 0.24	0.003
Wood	Lignin (%)	24.55 ± 1.67	21.89 ± 1.39	0.134
	FW/DW	2.23 ± 0.09	2.45 ± 0.12	0.100
	Vessel lumen area (μm^2^)	940.7 ± 53.0	1459.6 ± 81.1	<0.001
	Fiber double wall thickness (μm)	4.36 ± 1.32	3.44 ± 1.38	<0.001

Data are mean ± SE of 5 biological replicates. Independent two-sample *t*-tests were used to test the differences between the means for HN and LN plants. FW/DW = dry to fresh mass ratio.

**Figure 6:**
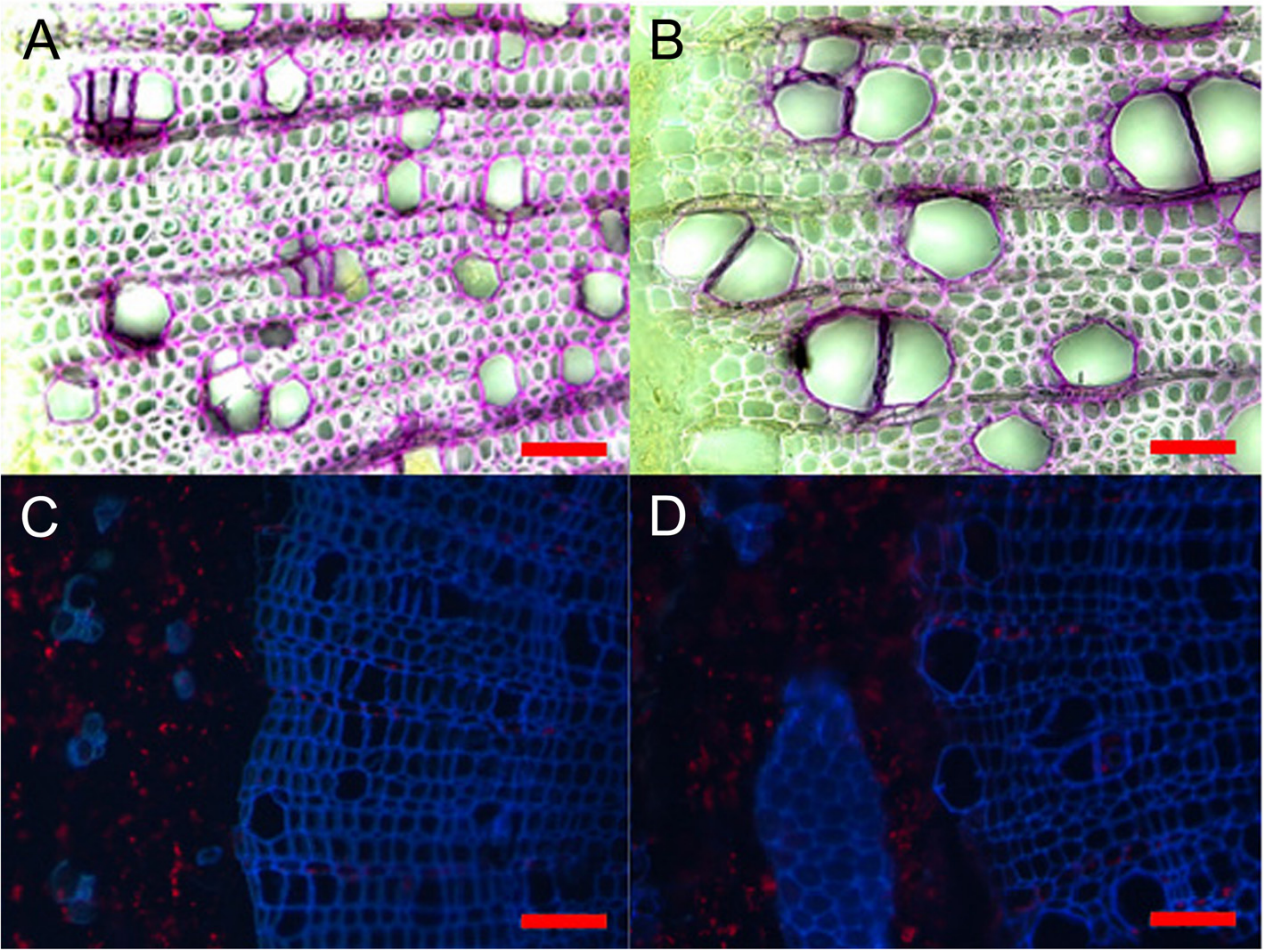
**Cross sections of in zone of secondary wood formation in stems of** ***P*** **.** ***trichocarpa*** **in response to low nitrogen (A, C) and high nitrogen (B, D) supply.** Lignin staining **(A, B)** of cross sections (50 μm thickness); autofluorescence of phenolics **(C, D)** in cross sections (10 μm thickness). The cross sections were taken in the second internode counted from the stem bottom. The scale bars correspond to 50 μm.

This finding was surprising because previous studies reported decreased lignin concentrations in wood of poplars grown with high N supply [34-36]. Therefore, we also determined lignin and soluble phenolics in lower stem parts. In concordance with earlier studies, we found decreased concentrations of lignin and phenolics in the developing xylem (Table 4). In wood from the lower stem segment, the differences were not significant, but those tissues had mainly been formed before the N treatment started. The autofluorescence of phenolics and lignin staining of the cross sections in the area of secondary wood formation supported the biochemical analyses and indicated reduced or delayed incorporation of secondary metabolites into the cell walls of the HN poplars (Figure 6).

### The linkage of N metabolism genes and N-responsive network

High N supply increased the N concentrations in all stem tissues including the EZ (Table 1). Still, “N metabolism” was not identified as a significant Mapman or GO term, but the term “amino acid metabolism” was significantly enriched on the basis of all DEGs (Additional file 2: Table S2). The list of N-related DEGs also contained nitrate reductase *PtNIA2.2* (Potri.005G172400), nitrite reductase *PtNIR1.1* (Potri.004G140800), various transporters for N-containing cargo [high affinity nitrate transporter *PtNRT2.7* (Potri.001G348300), ammonium transporter *PtAMT1-6* (Potri.009G045200), three amino acid transporters (Potri.010G226000, *PtAAT1.1* (Potri.012G131300), *PtLHT1.2* (Potri.001G335300), amino acid permease (Potri.003G103600)]. Among these genes, four were present in complex 1, of which two amino acid transporters (*PtAAT1.1*, *PtLHT1.2*) and a biosynthetic gene encoding a lysine-ketoglutarate reductase/saccharopine dehydrogenase (Potri.006G134200) were up-regulated, while a putative chorismate/shikimate dehydrogenase (Potri.014G135500) was down-regulated. Overall, the transcript abundances of the majority of the N-related genes (15/22) were decreased under HN compared with LN conditions (Additional file 2: Table S2).

## Discussion

### N-responsive cell wall formation in the poplar elongation zone

N plays a role as a signal to regulate plant gene expression for growth and development [48,49]. Changes in N availability are sensed by plants rapidly in time scales of minutes to days [50,51]. Therefore, the differences reported in the current study reflect alterations in the physiological status of long-term HN and LN acclimated plants. In agreement with previous studies [30-32,35,36] *P. trichocarpa* showed faster elongation of the upper stem internodes as well as increased radial growth at the stem base under HN compared with LN conditions. Furthermore, the anatomical alterations in response to high N in the wood were similar to those observed in previous studies, such as thinner cell walls, wider vessel lumina and decreased lignin staining intensity [30-33,35,36] corresponding to the negative correlation of growth and lignin [52]. An unexpected finding of our study was that the EZ of HN poplars contained increased concentrations of soluble phenolics and lignin as well as thicker cell walls compared with LN poplars. Apparently, during primary elongation growth the influence of high N on xylary elements is reversed compared to that during secondary growth. This notable result was supported by the identification of an underlying gene network. The network contained a tightly co-regulated subnet (complex 3) that was enriched in genes known to be involved in secondary cell wall formation. In addition to master regulators of secondary cell wall formation such as *MYB46* and *MYB103* [7], it integrated genes for cellulose synthase, for hemicellulose formation as well as laccases and peroxidases required for lignification. Moreover, complex 3 encompassed about one-third of the genes denominated as “core xylem gene set” in *Arabidopsis* [53] and contained various genes found to be preferentially expressed during secondary wall formation in wood of *P. tomentosa* [54]. In contrast to the remarkable overlap with the genes for secondary cell wall formation, we found only one common gene between complex 3 and the N-regulated genes earlier identified in the developing xylem of *Populus trichocarpa* × *deltoides* ([36], Additional file 5: Table S4). This observation further supports divergent regulation of cell formation in the EZ and the developing xylem that might have led to the striking differences in cell wall anatomy and biochemistry.

Although the overlap with complex 3 was low, we found that about 13% of the genes identified in the developing xylem of HN poplars by Plavcová et al. [36] (Additional file 5: Table S4) were also present in our total DEG list, including for example the aquaporin TIP1;3 and genes for amino acid metabolism. Plavcová et al. [36] speculated that enhanced aquaporin expression may be required for increased water uptake as a precondition to drive the strong expansion of the vessel lumina under high N. In concordance with this suggestion we found that the EZ of HN poplars contained higher water content and strongly enlarged vessel lumina compared with LN poplars.

In our study, the identified cell wall-related complex 3 may be considered as a “hub” because of its high connectivity. Hubs may constitute regulatory units [55]. Therefore, we inspected potential regulatory genes in complex 3 with connectivity to cell wall-related genes. In this context, *PtARAC2.1,* a poplar homolog to *AtRAC2/ROP7* was most notable. In Arabidopsis, the expression of *AtRAC2/ROP7* is developmentally limited to the late stages of xylem differentiation and coincides with the formation of secondary cell walls [56]. The AtRAC2/ROP7 promoter directs highly xylem-specific expression in *Arabidopsis*. In our subnet, *PtARAC2.1* (*AtRAC2*/*ROP7*, AT5G45970) had 32 edges and was highly connected with cell wall-related genes including three fasciclin-like genes (*PtFLA11.1*, *PtFLA14.7*, *PtFLA14.8*); four cellulose synthases [*PtCESA.2* (*CESA8*), Potri.002G257900 (*CESA4*), Potri.018G103900 (*CESA7*), Potri.011G069600 (*CESA8*)], genes for hemicellulose and xylan biosynthesis [Potri.006G131000 (*IRX9*), Potri.016G086400 (*IRX9*); Potri.003G100200 (*PRR1*)], lignin formation [Potri.016G112000 (*LAC4*)] and regulation [Potri.008G094700 (*KLCR2*); Potri.015G060100 (*COBL4*)]. All these genes were up-regulated in the EZ of HN compared with LN plants. *PtARAC2.1* is also present in the list of the “core xylem gene set” [53] and, thus, is a promising candidate to uncover the regulation of cell wall formation in poplar. Overall, the massive transcriptional regulation of regulatory and biosynthetic plant cell wall genes in the EZ suggests that high N supply initiates differences in cell wall formation at an early developmental stage.

### N-driven elongation growth is under stress

Our results revealed a co-expressed gene cluster (complex 1) with functional annotations for transcription factors, development, cell walls, stress and transport in the EZ of HN poplars. This complex apparently integrated processes of primary steps in cell wall formation (pectin) and phytohormone-regulated growth. For example, the observed enhancement of auxin-related gene transcript levels supports activation of cell division and enlargement in response to N. However, we also detected genes required for jasmonate biosynthesis and signalling (homologs to *JAR1, JAZ5, MYC2*). This was unexpected because jasmonate production is a response to wounding or herbivorous insects [57] and can induce plant stunting [58]. Growth exerts a strain on the enlarging tissues [59]. During extension growth the primary xylary elements are ruptured. We speculate that these processes may cause intrinsic lesions that could stimulate defence responses.

It was also conspicuous that *WRKY26* and *WRKY33* (regulation of heat-induced ethylene-dependent response of *Arabidopsis* [60]), *WRKY18* (activated downstream of a MAPK signalling pathway responding to pathogens [61,62]) and *WRKY40* (responses to abscisic acid (ABA) and abiotic stress [63]) were up-regulated. In concert with elevated transcript levels of a putative chitinase (*CHIV*), *PAD4*, *PR4, OSMOTIN 34* and the chaperone-like *DnaJ* family gene, these findings suggest that N-induced growth imposes stress on the tissues.

### N metabolism in the stem elongation zone

Nitrate reductase (*NIA2*), nitrite reductase (*NIR*) and the nitrate receptor/transporter *NRT1.1* are considered as sentinels of the nitrate response [64]. In poplar tissues *PtNRT1.1* has a low expression and therefore, cannot reliably be detected on microarrays [65]. Its expression is decreased under HN in the poplar EZ [66]. The expression of further poplar N-related genes *PtNIA2.2, PtNIR1.1* and *Potri.001G348300 (AtNRT2.7)* were suppressed, whereas several putative amino acids transporter were up-regulated in the EZ. This observation suggests that N assimilation does not play a major role in the EZ and suggests that the supply of the tissue is mainly achieved by the translocation and uptake of amino acids. Amino acids, mainly glutamine, are the principle long distance transport forms of nitrogen in poplars [27]. In the EZ we found increased expression of *PtLHT1.2*, a homolog to *Arabidopsis LHT1* (*LYSINE HISTIDINE TRANSPORTER1*). *AtLHT1* is a master switch directing the partitioning of glutamine during defence responses [67]. Here, *PtLHT1.2* was co-expressed with the *WRKY18* and *WRKY26* homologs, *PtAAT1.1* (AAA-type ATPase), Potri.016G071600 (late embryogenesis abundant protein) and *PtIFS1.42* (cytochrome P450, a putative gene of the brassinosteroid metabolism). This gene cluster could therefore coordinate growth processes with amino acid requirement. The molecular cross talk between these co-expressed genes remains still enigmatic. Further studies are needed to elucidate the causal links between N metabolites, their transport and growth regulation.

N assimilation takes mainly place in mature leaves, because it requires reducing power and carbon skeletons from photosynthesis as precursors for amino acid biosynthesis. It has often been reported that nitrogen and photosynthesis are positively related [68-70]. Contrary, N starvation generally resulted in decreased transcript levels of photosynthesis gene expression in Arabidopsis shoots [51]. It was, therefore, unexpected that genes for light reactions and the Calvin cycle were collectively suppressed in the EZ of HN plants (complex 2). Young stems are photosynthetically active [71,72], but the EZ is a sink tissue. Sink tissue rely on the import of carbohydrates and amino acids and respiration is stronger than photosynthesis [73]. This may be a reason why the photosynthetic genes were collectively suppressed in complex 2 of the EZ, whereas generally positive relationships between N supply and the expression of photosynthetic genes and photosynthetic activity exist [51].

## Conclusions

In the present study, we have identified genes involved in N-driven stem elongation growth in *P. trichocarpa.* Co-expression analysis extracted a network of DEGs with functional annotations to hormone metabolism, stress, transport, cell wall, and photosynthesis. The network uncovered three main complexes that represented functional units: Complex 1 integrated growth processes and stress suggesting that genes which have well established functions in abiotic and biotic stress are also recruited to coordinate growth strain. Complex 2 was enriched in genes with decreased transcript abundance and functionally annotated as photosynthetic hub. This finding underpins the complex relationship between photosynthetic processes and nitrogen. Complex 3 was identified as a hub for secondary cell formation because it connected well-known master regulators of secondary cell walls (e.g. *MYB46*) with genes related to the formation of cellulose, hemicelluloses, lignin and phenolics. Anatomical and biochemical analysis confirmed that N-driven growth resulted in early secondary cell wall formation in the elongation zone. In contrast to the EZ, secondary xylem at the stem base formed thinner cell walls with less lignin with high N supply. These results suggest that the influence of high N on cell wall deposition in xylary elements is reversed or shifted between secondary to primary growth. This finding may have practical implications because a reduction of the cellulose-to-lignin-ratio in the secondary xylem due to N fertilization affects the usability and economic value of wood as feedstock for biofuel production [3]. An important goal of future studies will be to elucidate the nitrogen-related regulation of the cell wall hub. This knowledge may open new perspectives on sustainable fertilization without negative consequences for wood composition.

### Availability of supporting data

The data sets supporting the results in this article are available in this article, in the additional files and in the ArrayExpress repository with the accession number E-MTAB-1483 (http://www.ebi.ac.uk/arrayexpress/experiments/E-MTAB-1483/).

## Additional files

Additional file 1: Table S1. N responsive gene list for the elongation zone. Additional file 2: Table S2. Annotated list of genes retrieved in the co-expression network. Additional file 3: Figure S1. A co-expression network of differentially expressed genes in the elongation zone of *Populus trichocarpa*. Additional file 4: Table S3. List of the most connected hub genes. Additional file 5: Table S4. Comparison of nitrogen responsive gene lists in the elongation zone (this study), the developing xylem [36] and the “core xylem gene set” [53].

## Abbreviations

ABA: Abscisic acid
DEG: Differentially expressed genes
EZ: Elongation zone
GO: Gene Ontology
HN: High nitrogen
JA: Jasmonic acid
JGI: The Joint Genome Institute
LN: Low nitrogen
N: Nitrogen
RMA: Robust Multiarray Averaging
SD: Standard deviation
