# Supplementary material for: Nitrogen-driven stem elongation in poplar is linked with wood modification and gene clusters for stress, photosynthesis and cell wall formation
Source: BMC Plant Biol. 2014 Dec 30;14:391. doi: 10.1186/s12870-014-0391-3 (PMC4302602; doi:10.1186/s12870-014-0391-3)
Supplement: Additional file 3: Figure S1. — A co-expression network of differentially expressed genes in the elongation zone of Populus trichocarpa. [file 12870_2014_391_MOESM3_ESM.pptx]

## Slide 1
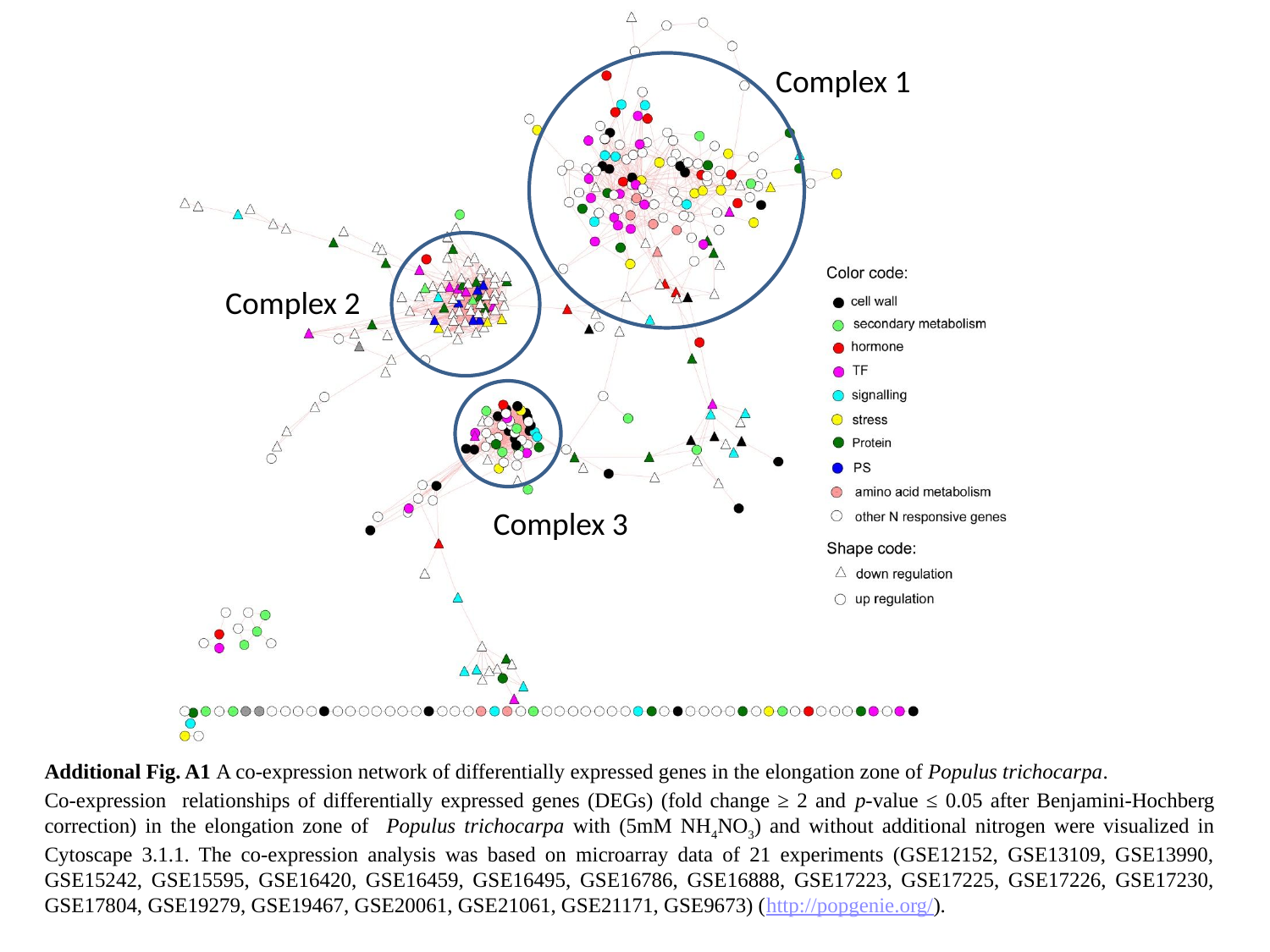

Complex 1
Complex 2
Complex 3
Additional Fig. A1 A co-expression network of differentially expressed genes in the elongation zone of Populus trichocarpa.
Co-expression relationships of differentially expressed genes (DEGs) (fold change ≥ 2 and p-value ≤ 0.05 after Benjamini-Hochberg correction) in the elongation zone of Populus trichocarpa with (5mM NH4NO3) and without additional nitrogen were visualized in Cytoscape 3.1.1. The co-expression analysis was based on microarray data of 21 experiments (GSE12152, GSE13109, GSE13990, GSE15242, GSE15595, GSE16420, GSE16459, GSE16495, GSE16786, GSE16888, GSE17223, GSE17225, GSE17226, GSE17230, GSE17804, GSE19279, GSE19467, GSE20061, GSE21061, GSE21171, GSE9673) (http://popgenie.org/).
